# An isolated rupture of the anterolateral ligament of the knee: A case report

**DOI:** 10.1097/MD.0000000000034259

**Published:** 2023-07-14

**Authors:** Anthony Elias El Alam, Joeffroy Naji Otayek, Joe Georges Ghanimeh, Jalal Mohamad El Karaaoui, Sahar Fawzi Semaan, Alfred Pierre Khoury

**Affiliations:** a Orthopedic Surgery, Lebanese American University Medical Center - Rizk Hospital, LAU Gilbert and Rose-Marie Chaghoury School of Medicine, Beirut, Lebanon; b Diagnostic radiology, Lebanese American University Medical Center - Rizk Hospital, LAU Gilbert and Rose-Marie Chaghoury School of Medicine, Beirut, Lebanon.

**Keywords:** anterolateral ligament, case report, knee, Segond

## Abstract

The anterolateral ligament (ALL) is a controversial ligament in the knee that may play a significant role in knee stability. It is difficult to identify on magnetic resonance imaging (MRI) imaging and is often injured in conjunction with other ligaments, such as the anterior cruciate ligament (ACL) and medial collateral ligament.

**Case presentation:**

This is a unique case of an isolated ALL tear in a 48-year-old woman who presented with severe left knee pain, swelling, and inability to bear weight during a yoga session. Physical examination showed swelling and tenderness at the lateral aspect of the femoral condyle, with increased pain on varus stress testing. Radiographs revealed normal osseous structures with the absence of traumatic bone lesions. MRI revealed an intact meniscus, cruciate, and collateral ligaments, but a rupture of the ALL at its femoral origin. Diagnosis of isolated ALL rupture of the left knee was made, and the patient was treated conservatively with icing, rest, and non-steroidal anti-inflammatory drugs. Physiotherapy was started 2 weeks post-injury, and return to sports was allowed at the sixth week. Upon last follow-up, the patient had excellent functional outcomes and was satisfied with the treatment. Physical examination showed a stable knee with negative Lachman and pivot shift tests. To the best of the authors’ knowledge, this is the first case of isolated ALL rupture to be reported.

**Discussion:**

The paper highlights the rarity of isolated ALL injuries and the difficulty in diagnosing them. Conservative treatment can be successful for isolated ALL injuries, with physiotherapy playing an essential role in rehabilitation.

In conclusion, isolated ALL injuries are rare and can be challenging to diagnose. Conservative treatment with physiotherapy can lead to successful outcomes. Further research is needed to understand the role of the ALL in knee stability and to determine optimal treatment options.

## 1. Introduction

The anterolateral ligament (ALL), a major component of the anterolateral corner of the knee, had created a controversy for a long time about its existence and its function. This ligament was first described by the French surgeon, Paul Segond, when he described an avulsion fracture of the anterolateral aspect of the proximal tibia as a result of a forced internal rotation.^[1]^ However it was not clear if it is related to a separate ligamentous structure or to fibers of the ilio-tibial band. It has then been suggested that the ALL plays a major role in the internal tibial rotation and hence in the rotational stability of the knee.^[2]^

The ALL originates from the lateral femoral condyle, anterior to the lateral collateral ligament with its posterior fibers seen interposed proximally with the lateral collateral ligament. Then, it courses inferiorly and shows close connection with the body of the lateral meniscus and attaches distally to the anterolateral aspect of the tibia, posterior to Gerdy tubercle.^[2]^

Different studies have suggested that the injury of the ALL is a part of a multi-ligamentous injury, with the anterior cruciate ligament (ACL) being the most commonly affected ligament with ALL injury.^[3]^ Other studies show isolated Segond fracture without any other ligamentous injury.^[4]^ In this paper, we present the case of a 48-year-old woman with isolated ALL tear following a varus stress on the knee.

## 2. Case presentation

**Figure 1.**
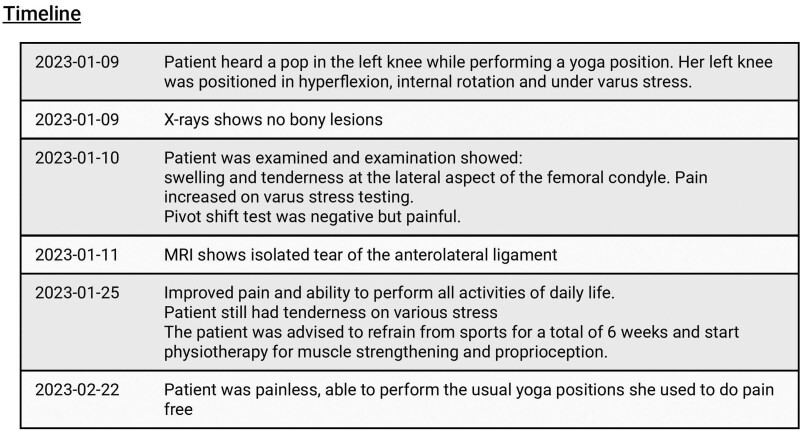
Timeline of the case presentation.

Timeline of the case presentation is shown in Figure 1.

A 48-year-old white woman was brought to our department with severe left knee pain, swelling and inability to bear weight. One day prior to presentation she heard a pop in the left knee while performing a yoga position. Her left knee was positioned in hyperflexion, internal rotation and under varus stress.

Patient reports no previous medical or surgical history.

Physical examination showed a young active and fit woman. The patient had swelling and tenderness at the lateral aspect of the femoral condyle. Pain increased on varus stress testing. Lachman, McMurray, anterior drawer, posterior drawer tests and sag sign were all negative. Pivot shift test was negative but painful.

Otherwise, the patient had no known medical or surgical history. She did not report any symptoms or knee related complaints previous to the injury. The patient was living a regular active life, doing sports (Yoga and Jogging) 3 to 4 times per week.

**Figure 2.**
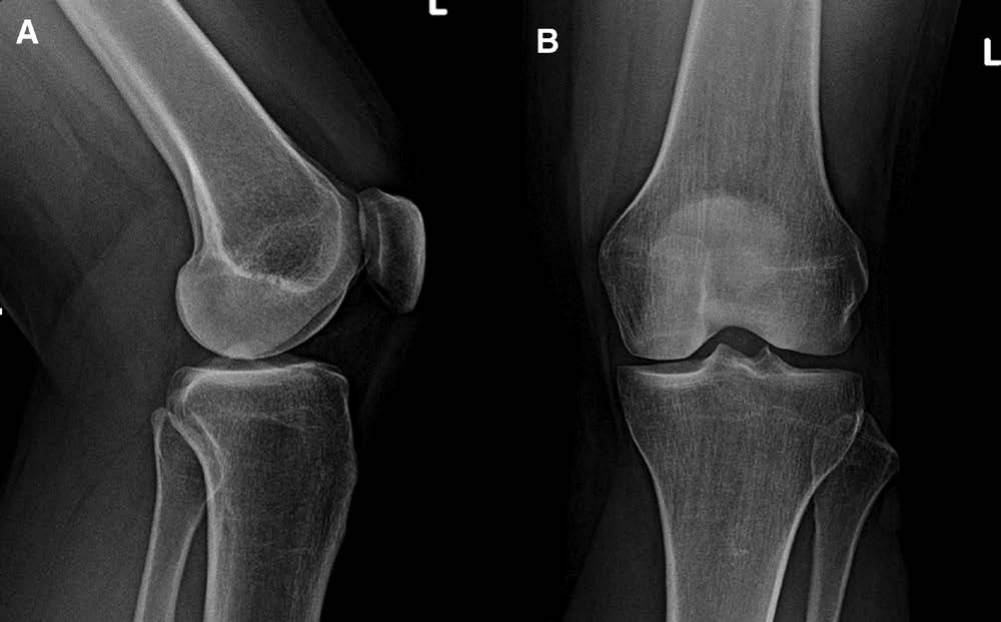
Lateral (A) and anteroposterior (B) knee radiographs showing no traumatic changes.

Left knee radiographs showed absence of traumatic bone lesions, with normal alignment and preservation of the femorotibial joint space (Fig. 2).

**Figure 3.**
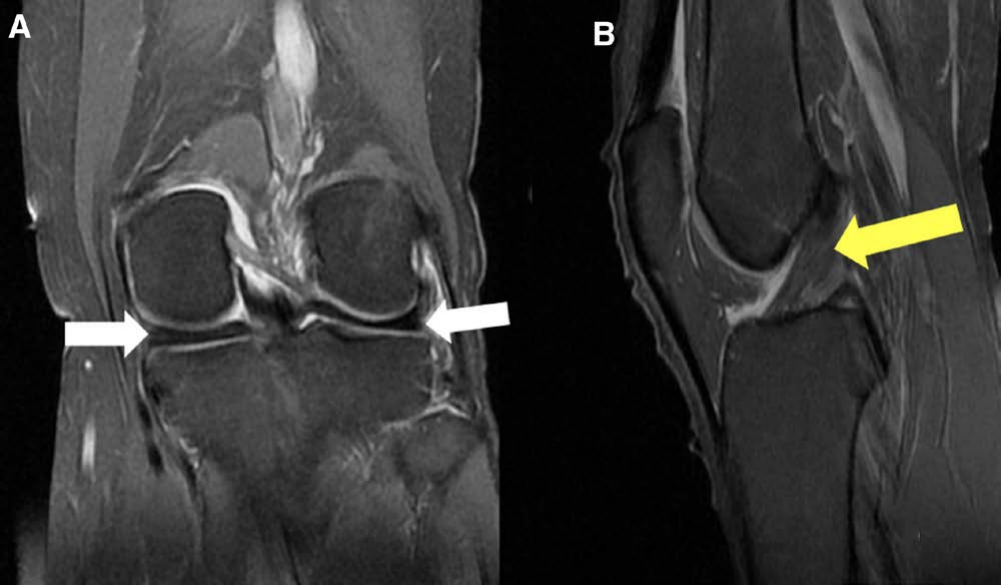
Coronal (A) and Sagittal (B) MRI cuts demonstrating intact menisci (white arrows) and ACL (yellow arrow). ACL = anterior cruciate ligament, MRI = magnetic resonance imaging.

Magnetic resonance imaging (MRI) was performed and showed intact menisci, cruciate, and collateral ligaments (Fig. 3).

**Figure 4.**
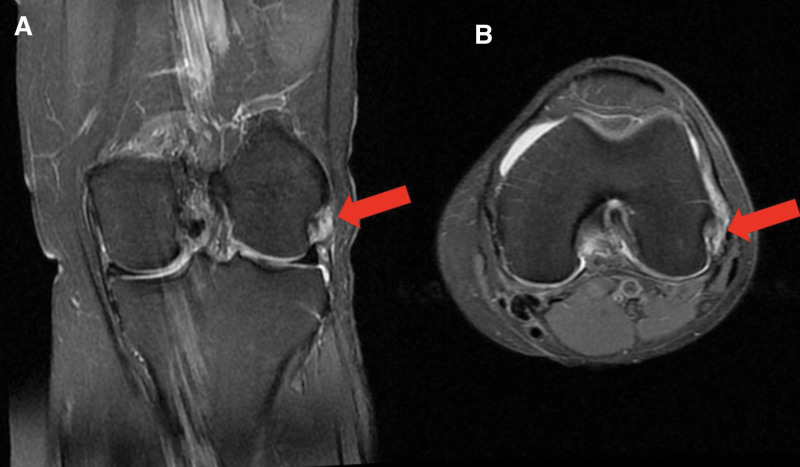
Coronal (A) and axial (B) MRI cuts demonstrating rupture of the anterolateral ligament at its femoral origin (red arrows). MRI = magnetic resonance imaging.

Rupture of the ALL at its femoral origin with edema and fluid seen at this level (Fig. 4).

The diagnosis of isolated ALL rupture of the left knee was made.

The patient was treated with rest, icing and non-steroidal anti-inflammatory drugs for 2 weeks, with a follow up 2 weeks later. In the meantime, ambulation and activity was allowed as tolerated.

Upon follow up (2 weeks) she reports improved pain and ability to perform all activities of daily life. On physical examination she still had tenderness at the lateral femoral condyle and on varus stress test.

The patient was advised to refrain from sports for a total of 6 weeks and start physiotherapy for muscle strengthening and proprioception.

At 6 weeks follow up, patient was painless, able to perform the usual yoga positions she used to do pain free.

With the patient reporting great improvement progressive return to sports was recommended.

## 3. Discussion

The ALL is the main component of the anterolateral corner of the knee. It arises from the lateral femoral epicondyle and insert at the anterolateral tibia, just proximal and posterior to the Gerdy tubercle.^[2,5–10]^

This ligament was first described by the French surgeon Paul Ferdinand Segond during his cadaver studies as a pearly thick resistant band that showed extreme tension during forced internal rotation of the knee.^[1]^ A fracture at the tibial zone of insertion of the ALL was also described in 1878 in response to an internal rotation stress to the knee.^[5]^ Though the works of Wood et al,^[11]^ Goldman et al,^[12]^ and Hess et al^[13]^ have built the belief that Segond fractures are pathognomonic of an ACL tear, Claes in 2014 demonstrated that the ALL is in fact the structure responsible for this avulsion fracture.^[14]^

Identification of the ALL ligament on MRI imaging is considered a challenge due to anatomical variabilities, MRI magnetic strength, and variable expertise in ALL evaluation. Helito et al were able to identify at least a portion of the ligament in 97.8% of the cases with however complete identification in only 71.7% of the cases, while using a 1.5T MRI.^[15,16]^

It has been reported that a torn ALL may be the reason behind a positive pivot shift test in a patient with an intact or a reconstructed ACL.^[17,18]^ Moreover, any ALL lesion, whether it is a sprain, rupture or avulsion is highly associated with other ligamentous knee injuries. The particularity of the present case resides in the rarity of an isolated ALL injury.

A study performed by Luke Lintin et al on the different patterns of injuries of the ALL in the setting of acute trauma, showed a high association with ACL injury followed by other ligamentous or meniscal injuries. In this retrospective study, 200 MRI were taken in patient with knee trauma, showing 75 ALL injuries (tears and sprains). An associated ACL injury was seen in 81% of the cases, medial collateral ligament in 68% and popliteofibular ligament in 38%. However, no cases of isolated ALL injury were reported. Some of the patients who presented an ALL injury without ACL tear had signs of transient patellar dislocation with however no clear association described previously.^[19]^

To the best of our knowledge, this is the first reported case of an isolated ALL rupture with no associated Segond fracture nor other ligamentous knee injuries.

Due to the lack of evidence concerning the treatment of ALL injuries, especially when isolated, a watch-and-see approach was initiated for the present case. The patient was treated conservatively with icing, rest and non-steroidal anti-inflammatory drugs. Physiotherapy for muscle strengthening and proprioception was started 2 weeks after the injury to allow for pain to resolve, followed by progressive return to sporting activity as tolerated at the 6th week from the initial injury.

Patient reported improvement of her symptoms. Her physical examination at 3 months follow up showed a stable knee with a negative Lachman and pivot shift test. Thus, no further treatment was mandated.

## 4. Patient perspective

Patient was very satisfied, pain free and has returned to sporting activities.

## 5. Conclusion

Varus stress trauma to the knee has the potential to cause ALL injury, even in the absence of associated ligamentous or bony damage.

Therefore, it is crucial for physicians to consider this possibility and perform a thorough evaluation to rule out any significant lesions of the cruciate ligaments, menisci, and collateral ligaments. However, if no other significant lesion is detected, conservative management may be attempted and seems to yield good functional results.

## Acknowledgments

We would like to express our gratitude to the patient for her willingness to participate in this case report. Their cooperation and openness throughout the process have been invaluable in our understanding of this medical condition. We also thank the healthcare professionals who were involved in the patient care. Finally, we extend our appreciation to our colleagues for their feedback and support in the preparation of this report.

## Author contributions

**Conceptualization:** Anthony El Alam, Joe Ghanimeh.

**Investigation:** Joeffroy Otayek, Joe Ghanimeh, Jalal El Karaaoui.

**Methodology:** Alfred Khoury.

**Resources:** Jalal El Karaaoui, Sahar Semaan, Alfred Khoury.

**Supervision:** Sahar Semaan, Alfred Khoury.

**Validation:** Sahar Semaan, Alfred Khoury.

**Writing – original draft:** Anthony El Alam, Joe Ghanimeh.

**Writing – review & editing:** Anthony El Alam, Joeffroy Otayek, Alfred Khoury.
